# Noncoding RNAs in Tumor Epithelial-to-Mesenchymal Transition

**DOI:** 10.1155/2016/2732705

**Published:** 2016-02-16

**Authors:** Ching-Wen Lin, Pei-Ying Lin, Pan-Chyr Yang

**Affiliations:** ^1^Institute of Biomedical Sciences, Academia Sinica, Taipei 11529, Taiwan; ^2^National Center of Excellence for Clinical Trials and Research Center, Department of Medical Research, National Taiwan University Hospital, Taipei 10043, Taiwan; ^3^Department of Internal Medicine, National Taiwan University Hospital and College of Medicine, National Taiwan University, Taipei 70101, Taiwan

## Abstract

Epithelial-derived tumor cells acquire the capacity for epithelial-to-mesenchymal transition (EMT), which enables them to invade adjacent tissues and/or metastasize to distant organs. Cancer metastasis is the main cause of cancer-related death. Molecular mechanisms involved in the switch from an epithelial phenotype to mesenchymal status are complicated and are controlled by a variety of signaling pathways. Recently, a set of noncoding RNAs (ncRNAs), including miRNAs and long noncoding RNAs (lncRNAs), were found to modulate gene expressions at either transcriptional or posttranscriptional levels. These ncRNAs are involved in EMT through their interplay with EMT-related transcription factors (EMT-TFs) and EMT-associated signaling. Reciprocal regulatory interactions between lncRNAs and miRNAs further increase the complexity of the regulation of gene expression and protein translation. In this review, we discuss recent findings regarding EMT-regulating ncRNAs and their associated signaling pathways involved in cancer progression.

## 1. Introduction

Epithelial-to-mesenchymal transition (EMT) is a critical step in both embryonic development and tumor metastasis. EMT is composed of serial phenotypic changes through which epithelial cells lose their apical-basal polarity and tight cellular adhesions, while acquiring protease-producing properties that increase cell motility [1]. EMT is a well-recognized process in tumor metastasis through which tumor cells seed and colonize areas distant from their primary sites. The process of EMT is sophisticatedly regulated and requires the acquisition of variable genetic alterations among tumor cells and their microenvironment [2, 3]. Important cellular components of the tumor microenvironment (TME) include tumor-infiltrating immune cells, cancer-associated fibroblasts, and endothelial cells. In addition, hypoxic conditions, which alter the composition of extracellular matrix (ECM), cytokines, chemokines, and growth factors, are critical in the development of EMT [4, 5].

Among important TME-associated cytokines are members of the transforming growth factor-*β* (TGF-*β*) family, which paradoxically suppress tumor metastasis in early-stage cancers but drive the metastatic process in advanced disease. TGF-*β* signaling initiates EMT by activating EMT-inducing transcription factors (EMT-TFs), such as Snail/Slug, zinc-finger E-box-binding homeobox 1/2 (ZEB1/2), basic helix-loop-helix (bHLH) protein, E47, and Twist, or by transcriptionally repressing epithelial-specific genes via members of the histone deacetylase (HDAC) family [6–10]. Epithelial-specific genes, such as E-cadherin (*CDH1*), zona occludens 1 (*ZO-1*), and occludin (*OCLN*), are substantially downregulated at the transcriptional level during the EMT process [11–13]. Notably, promoter regions of* CDH1* and* OCLN* genes contain EMT-TF binding sites, termed E-boxes.* CDH1* and* OCLN* are frequently downregulated in high-grade malignancies with poor clinical outcomes [14–17], whereas mesenchymal markers, such as N-cadherin, vimentin, fibronectin, and *α*-smooth muscle actin (*α*-SMA), are upregulated [18]. Dynamic expression of these proteins results in alterations in cytoskeleton arrangements and cellular polarity, as well as changes in the ability of cells to degrade ECM.

Recent cancer genomic studies have identified numerous RNAs that do not encode proteins. These noncoding RNAs (ncRNAs), including snRNAs, snoRNAs, rRNAs, tRNAs, piRNAs, microRNAs (miRNAs), and long noncoding RNAs (lncRNAs), regulate biological functions through interactions between their specific structural domains and DNA, RNA, or proteins [19]. Of these ncRNAs, miRNA and lncRNAs have been found to serve as important gene expression regulators that fine-tune cell transcriptomes and adjust proteomes in response to extracellular stimulation [20]. In addition, these noncoding RNAs could be transported from primary site to another cell or distant organ through extracellular vesicles and alter the gene expression profile as well as their morphology and functions within the target sites [21, 22]. Furthermore, mutations and dysregulations of miRNAs and/or lncRNAs are associated with a diverse array of human diseases, including cancer [23–27].

**Figure 1:**
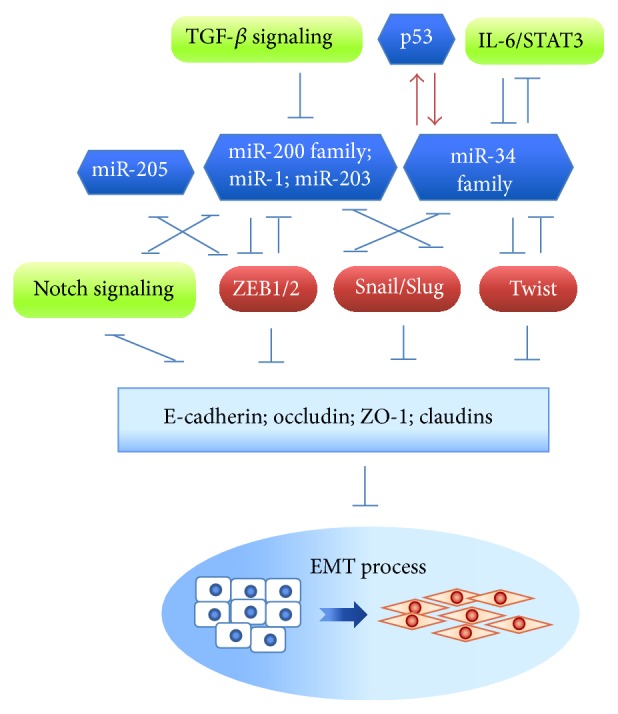
The reciprocally regulatory feedback loop between miRNAs and EMT-TFs that are involved in EMT. miRNAs form regulatory networks with EMT-TFs and EMT-associated signaling pathways that individually or cooperatively modulate EMT. EMT-suppressing miRNAs, such as the miR-200 family, miR-1, miR-203, and the miR-34 family (in blue), reciprocally suppress EMT-TFs (ZEB1/2, Snail/Slug, and Twist) and consequently downregulate the expression of epithelial markers (E-cadherin, occludin, ZO-1, and claudins). This negative feedback loop can be broken by TGF-*β* or IL-6/STAT3 signaling, and p53.

In this review, we discuss recent findings on the roles of miRNAs and lncRNAs in regulating EMT-TFs (Tables 1 and 2). We also discuss the multilayered regulatory circuits among miRNAs, lncRNAs, and protein-coding genes that are associated with cancer EMT (Figure 1).

## 2. EMT-Related Signaling Pathways and the Tumor Microenvironment

The TME is a niche composed of various growth factors secreted by tumor cells or adjacent tissues, cytokines released by lymphoid cells, molecular components of the ECM, and intratumor hypoxia. The expression of EMT-TFs in cancer cells can be turned on in response to changes in the extracellular microenvironment. Signaling pathways, including those mediated by TGF-*β*, bone morphogenetic protein (BMP), Wnt, Notch, integrin, epidermal growth factor (EGF), fibroblast growth factor (FGF), platelet-derived growth factor (PDGF), and sonic hedgehog (SHH), are overactivated during carcinogenesis [28, 29]. In addition, tumor hypoxia is responsible for the expression of a subset of EMT-TFs and activation of a category of EMT-related signaling pathways. Here, we discuss several signaling pathways that participate in the initiation of cancer EMT.

### 2.1. TGF-*β* Signaling Pathway

TGF-*β* signaling is a core pathway that tightly controls the process of cell proliferation and EMT during organ development, tissue fibrosis, and cancer progression [30]. This signaling pathway is typically initiated by ligands belonging to the TGF-*β* superfamily, which includes three isoforms of TGF-*β* (TGF-*β*1, TGF-*β*2, and TGF-*β*3) and six isoforms of BMP (BMP2–7). These ligands are expressed and secreted in different cellular contexts and in response to various stimuli [31]. TGF-*β* receptors are single-pass serine/threonine kinases that exist in different isoforms, including seven Type I (TGF-*β*RI) and five Type II (TGF-*β*RII) receptors that can form homo- or heterodimers. Combinatorial dimerization enables TGF-*β* receptors to differentially activate intracellular signaling pathways that are broadly distinguished by their SMAD dependence or independence. In response to phosphorylation of TGF-*β* receptors, a SMADs ternary complex, composed of SMAD2/3, R-SMAD and SMAD4, forms and translocates from the cytoplasm to the nucleus [32].

Several EMT-TFs, including members of the ZEB family, Snail/Slug and Twist, are transcriptionally upregulated in cancer cells by TGF-*β* signaling through conserved response elements on the promoters of the corresponding genes [10, 33–36]. TGF-*β*-SMADs was also shown to indirectly induce expression of EMT-TFs by enhancing the expression of its downstream effector, high mobility group A2 (HMGA2) [37, 38]. Activated TGF-*β* signaling is sustained by an autocrine loop, which in turn reinforces the EMT process [39–41]. Furthermore, Snail and SMAD3/4 form a transcriptional repressor complex, which synergistically suppresses the expression of coxsackie and adenovirus receptor (*CAR*),* OCLN*, and* CDH1*, and thus promotes EMT [42]. These results suggest the importance of TGF-*β* signaling in cancer EMT and its potential to serve as a therapeutic target.

### 2.2. Wnt, Notch, and MAPK Signaling Pathways

The Wnt signaling pathway is an important regulator of EMT-TF expression and the EMT process. WNT couples with the membrane protein Frizzled and low-density lipoprotein receptor (LRP), promoting translocation of *β*-catenin from the cytoplasm to the nucleus. In the nucleus, *β*-catenin acts as a coactivator of TCF/LEF1 (T cell factor/lymphoid-enhancing factor-1) and upregulates the transcription of* SNAIL1/2* and* TWIST*, which in turn repress E-cadherin [43–45].

Notch signaling is activated by cell-cell contact. Interactions between JAG1/2 (Jagged-1/2), Notch ligand, and Notch receptors facilitate nuclear translocation of the Notch intracellular domain (NICD), which subsequently activates Notch effector genes [46]. Notch signaling not only enhances* SNAIL* transcription but also enhances SNAIL1/2 function through upregulation of hypoxia-inducible factor 1*α* (HIF-1*α*), thereby promoting tumor invasion and/or metastasis [47, 48].

Additional pathways are also involved in cancer EMT. For example, hepatocyte growth factors (HGFs) and insulin-like growth factor-1 (IGF-1) upregulate expression of* SNAIL* and* ZEB1*, respectively, through the mitogen-activated protein kinase (MAPK) pathway [49–51]. Collectively, these observations suggest that EMT-TF regulatory circuits are tightly controlled.

### 2.3. Tumor Microenvironment and Hypoxia

Hypoxic microenvironments, defined as those with a pO_2_ level less than 10 mmHg, trigger signaling cascades and immune responses that drive cancer progression [52, 53]. The hypoxic microenvironment contributes to the immune escape of tumors as well as tumor neovascularization and also promotes EMT [4]. Tumor hypoxia-dependent signaling is predominantly mediated by hypoxia-inducible factors (HIFs)—important protein complexes that regulate tumor progression and metastasis [52]. HIFs, which consist of an unstable *α*-subunit and a stable *β*-subunit [54], bind to promoters of target genes that contain the hypoxia response element (HRE) and promote the recruitment of transcriptional coactivators. In HIF-1*α*-mediated canonical hypoxia signaling, expression levels of Twist, Snail, ZEB1, and E12/E47 are upregulated [55, 56].

Studies have shown that a hypoxic TME contributes to the stabilization of HIF-1*α*, which functions to activate TGF-*β* signaling [57, 58]. TGF-*β*, in turn, assists in the maintenance of HIF-regulated vascular homeostasis and angiogenesis [59, 60]. The promoter region of* VEGF* (vascular endothelial growth factor), encoding a secretory factor involved in vasculogenesis and angiogenesis, harbors both HIF-1*α* and SMAD binding sites, suggesting the possibility that both hypoxia and TGF-*β* signaling pathways regulate* VEGF* expression [61]. The positive feedback loop between HIF-1*α* and TGF-*β* functions in the regulation of cancer EMT and angiogenesis [62].

The TME-associated HIF-1*α*-mediated hypoxia pathway also regulates cancer EMT through Notch signaling [48, 63, 64]. It has been shown that interaction between NICD and HIF-1*α* increases the expression of Snail and Slug, which enhance cancer invasion and migration [48, 65]. In addition, a hypoxic TME augments the nuclear translocation of *β*-catenin, which promotes activation of Wnt signaling [66]. Collectively, these findings demonstrate that a hypoxic TME acts as a driving force for cancer EMT, both directly, through stabilization of HIFs, and indirectly, through paracrine/autocrine stimulation.

### 2.4. Chemoresistance

Recent findings by* in vivo* mesenchymal lineage tracing showed that EMT might not be essential for tumor metastasis, and interestingly the phenotype of EMT in tumor cells was resistant to CTX (cyclophosphamide) and gemcitabine treatment [67, 68]. Despite the fact that there may be other EMT-inducing factors function to compensate for the genes that were manipulated in these studies, the discovery of EMT tumors displayed chemoresistance may provide a new insight for developing novel therapy targeting tumor metastasis.

## 3. miRNAs and EMT

miRNAs are a group of small (~22 nucleotides) ncRNAs that mediate destabilization and translational suppression of downstream RNAs at the posttranscriptional level. The expression of miRNAs can be ubiquitous or context-specific (e.g., during development or within certain tissues). miRNAs participate in a broad range of physiological functions. Therefore, miRNA dysregulation may break the harmony of normal genetic activity and result in a diverse array of diseases, including cancer. Recent studies have revealed that more than 50% of miRNAs are dysregulated in human cancer. Moreover, prognostic and predictive miRNA signatures have been reported in different types of cancer [125–127].

miRNAs serve both positive and negative roles in regulating cancer EMT (Figure 1 and Table 1) [128]. The regulation of miRNAs is complicated, as highlighted by the fact that some miRNA targets can, in turn, regulate the expression of miRNAs, forming regulatory loops. Two EMT-related regulatory feedback loops formed by miRNAs and EMT-TFs will be discussed here: the miR-200 family and ZEB1/2, and miR-203/miR-34 and Snail/Slug.

### 3.1. Reciprocal Regulation between miRNAs and EMT-TFs: The miR-200 Family and ZEB1/2

The miR-200 family consists of five members, including miR-200a/b/c, miR-429, and miR-141, all of which contain a similar seed sequence that targets a large common subset of genes [129]. The miR-200 family directly suppresses ZEB1/2 translation, consequently upregulating the expression of E-cadherin and maintaining an epithelial cellular morphology [69, 129, 130]. Conversely, ZEB1 has been shown to strongly promote tumorigenesis and cancer metastasis by inhibiting miR-200c and miR-203 transcription. These data suggest that the miR-200 family and ZEB1/2 form a negative regulatory feedback loop [73].

In pancreatic and breast cancer models, the miR-200 family is reported to suppress Notch-mediated ZEB1 activation by directly targeting the Notch coactivators MAML2 and MAML3 and the Notch ligand, JAG1 [70]. In lung cancer, miR-200 and GATA binding protein 3 (GATA3), a direct downstream target of Notch involved in lung cancer metastasis, have been shown to mutually inhibit each other. This inhibitory loop between miR-200 and GATA3 is perturbed by the Notch ligand, JAG2 [131]. These results suggest that Notch signaling broadens the spectrum of the miR-200/ZEB1 negative feedback loop in regulating cancer EMT and metastasis.

It has also been reported that miR-200 is associated with the reverse EMT process—mesenchymal-epithelial transition (MET)—in prostate cancer. miR-200 and miR-1 directly target the* SLUG* 3′-UTR (untranslated region), and Slug in turn inhibits miR-200/miR-1 expression [71]. In addition, prolonged TGF-*β* signaling increases miR-200 promoter methylation and leads to miR-200 suppression. This suggests that the induction and maintenance of a mesenchymal state require autocrine TGF-*β* signaling to sustain expression of EMT-TFs and inhibition of the miR-200 family [132, 133].

### 3.2. Reciprocal Regulation between miRNAs and EMT-TFs: miR-203/miR-34 and Snail/Slug

Similar to the ZEB/miR-200 negative feedback loop, Snail together with the miR-34 family (miR-34a, miR-34b, and miR-34c) and miR-203 constitutes another negative feedback loop. This negative feedback loop regulates epithelial plasticity [75]. Snail and miR-34a/b/c control ZNF281/ZBP-99, a Krüppel-type zinc-finger domain-containing transcription factor, acting as an integral component of an EMT-related feed-forward loop [134]. A negative feedback loop between miR-203 and Snail controls the dynamic transition between epithelial and mesenchymal phenotypes [135, 136]. miR-203 has been shown to suppress Slug expression in breast cancer cells, whereas TGF-*β*-mediated Slug activation reciprocally downregulates miR-203 expression [136, 137].

A feedback loop also exists between p53 and miR34. The tumor-suppressor p53 upregulates the expression of miR-34, which subsequently suppresses EMT. Mutated p53 proteins, in contrast, are unable to induce miR-34 expression, thus shifting the equilibrium toward a mesenchymal phenotype [76, 138–140]. In addition, the p53/miR-34 axis suppresses Wnt signaling, both in development and during cancer progression [138].

These results illustrate how miRNAs create networks that connect different EMT-associated signaling pathways. EMT-related signaling not only upregulates EMT-TFs but also suppresses miRNAs; this, in turn, breaks down miR-200/ZEB and/or miR-203/Snail/Slug feedback loops and facilitates cancer EMT (Figure 1).

### 3.3. Other EMT-Related miRNAs

Several other miRNAs are reported to be involved in EMT (Table 1). For example, miR-10b is transcriptionally upregulated by Twist and induces tumor invasion and metastasis in breast cancers by targeting homeobox D10 (HOXD10) [87]. miR-9 directly targets E-cadherin mRNA, resulting in activation of *β*-catenin signaling, which promotes EMT and metastasis [78]. miR-9 upregulation has also been observed in* c-myc*-induced mouse mammary tumors [141]. In addition, miR-9 has been shown to downregulate leukemia inhibitory factor receptor (LIFR). LIFR suppresses breast cancer metastasis by activating the Hippo kinase cascade, which in turn results in YAP (YES-associated protein) inactivation [79].

A recent study revealed that miR-9-3p negatively regulates the expression of TAZ, a YAP homolog [142]. A recent study by our laboratory also showed that miR-135b increases the levels of nuclear TAZ by directly suppressing multiple components of the Hippo pathway, including LATS2 (large tumor-suppressor kinase 2), MOB1B (Mps one binder 1b), NDR2 (nuclear-Dbf2-related 2), and *β*-TrCP (*β*-transducin repeat-containing protein) [80]. Notably, miR-135b expression level, LATS2 protein, and nuclear TAZ protein levels correlate with disease prognosis in non-small-cell lung cancer patients [80]. Furthermore, YAP was found to physically interact with p72, a RNA helicase that plays roles in miRNA processing [143]. These results may suggest how the Hippo pathway suppresses proliferation in response to contact inhibition.

### 3.4. miRNAs Involved in Hypoxia-Induced EMT

Some miRNAs are involved in hypoxia-induced EMT. Expression of miR-205 and miR-124, which regulate EMT by targeting ZEB1/2 and MMP2 (matrix metallopeptidase 2), respectively, is suppressed by hypoxia [69, 144, 145]. Hypoxia also downregulates the expression of miR-34a, which acts as a suppressor of Snail and ZEB1. miR-34a suppression results in upregulation of Notch1 and JAG1, and activated Notch signaling promotes EMT [146]. Forced expression of miR-34a under conditions of hypoxia not only reduces Notch1 and JAG1 expression but also abolishes Snail expression, suggesting that the interplay between hypoxia and Notch signaling is important in EMT modulation (Figure 1).

## 4. Long Noncoding RNAs and EMT

lncRNAs are RNA transcripts longer than 200 nucleotides that do not encode proteins [147]. The FANTOM project revealed that, in mammals, the number of lncRNAs is at least four times that of protein-coding RNAs [148, 149]. Although the functions of lncRNAs are largely unknown, accumulating evidence suggests that they are key regulators of a number of important biological processes, possibly exerting tissue-specific imprinting patterns and functioning in embryogenesis, development, lineage differentiation, tumorigenesis, and EMT regulation [150–152].

**Figure 2:**
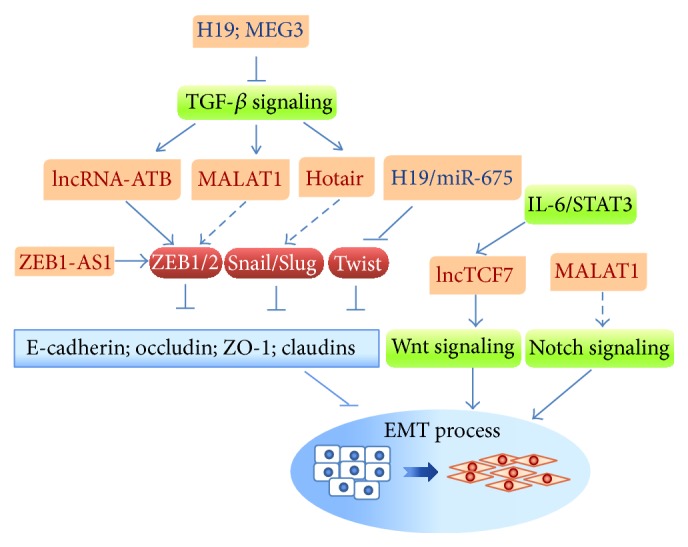
The reciprocally regulatory feedback loop between lncRNAs and EMT-TFs that are involved in EMT. lncRNAs form regulatory networks with EMT-TFs and EMT-associated signaling pathways that individually or cooperatively modulate EMT. The EMT-suppressive lncRNAs, H19 and MEG3, can downregulate TGF-*β* signaling. H19, lncRNA-ATB, and ZEB-AS1 promote EMT-TFs through direct or indirect regulation. In addition, H19 is reported to possess a controversial ability to downregulate Twist though its intergenic miRNA, miR-675. TGF-*β* and IL-6/STAT3 signaling pathways also promote activity of the lncRNAs, Hotair, lncTCF7, and MALAT1, and thus crosstalk with Notch signaling and Wnt signaling, to modulate EMT process. Dysregulation of these miRNAs and lncRNAs may lead to tumor progression.

Recent studies have shown that lncRNA dysregulation is associated with cancer progression [153]. lncRNAs may interact with their nearby protein-coding genes, for example,* TAL1, SNAIL, SLUG,* and the master regulator of hematopoiesis, SCL/TAL1 (T Cell Acute Lymphocytic Leukemia 1), as evidenced by the fact that depletion of certain lncRNAs results in upregulation of these genes [154]. In addition, some lncRNAs encompass miRNA binding sites. These lncRNAs function as ceRNAs (competing endogenous RNA) that antagonize miRNAs. lncRNAs may positively or negatively regulate EMT-associated proteins and miRNAs, as discussed below (Figure 2 and Table 2).

### 4.1. lncRNAs Regulate EMT through EMT-TFs

Most lncRNAs act as transcription inducers or form RNA-protein complexes that promote expression of EMT-associated genes at transcriptional or posttranslational levels [105]. The lncRNA, ZEB1-AS1, was found to be frequently upregulated in hepatocellular carcinoma (HCC) [103]. ZEB1-AS1, whose transcription locus is close to* ZEB1*, increases* ZEB1* promoter activity through an unknown mechanism. ZEB1, in turn, suppresses proteins that maintain the epithelial phenotype, such as E-cadherin, ZO-1, and occludin [103]. Thus, targeting ZEB1-AS1 may inhibit ZEB1-related EMT.

Expression of lncRNA-ATB, another lncRNA that upregulates ZEB1/2 expression and functions as a ceRNA, is enhanced by TGF-*β*. lncRNA-ATB promotes a metastatic cascade by competitively binding to members of the miR-200 family, thereby attenuating the inhibitory function of miR-200 on ZEB1/2. At the same time, lncRNA-ATB upregulates interleukin-11 (IL-11) and activates IL-11/STAT3 (signal transducer and activator of transcription 3) signaling, which enables cancer cell colonization [104].

lncRNA-HIT, a HOXA transcript induced by TGF-*β*, is a newly identified lncRNA upregulated by TGF-*β* that also mediates TGF-*β*-induced EMT [105]. It has been found that lncRNA-MEG3 associates with a PRC2 (polycomb repressive complex 2) complex through interactions with EZH2 (enhancer of zeste 2 polycomb repressive complex 2 subunit). lncRNA-MEG3 is recruited to a GA-rich sequence in target genes, forming a RNA-DNA triplex structure that regulates expression of the TGF-*β* receptor genes,* TGFBR1* and* TGFB2*, as well as* SMAD2* [106]. lncRNA-Hh, activated by Twist at the transcriptional level, directly targets GAS1 (growth arrest-specific 1) and activates hedgehog signaling [103]. The Twist/lncRNA-Hh signaling cascade enhances the stemness property of cells, suggesting a connection between EMT and stemness [107].

### 4.2. lncRNAs Regulate EMT through Their Interplay with miRNAs

EMT is a tightly controlled physiological and pathological process. Not only proteins but also lncRNAs and miRNAs are involved in fine-tuning EMT regulation. lncRNAs may serve as ceRNAs, which act as molecular “sponges” to regulate the harmony of miRNA pools and the biological signaling regulated by them. Interestingly, ceRNAs absorb target miRNAs without altering their total amount. Therefore, it is important to note that biological functions of miRNAs are simply determined not only by their measured abundance but also by their interactions with lncRNAs. lncRNAs are thus attractive therapeutic targets in miRNA-mediated diseases. Three lncRNAs—H19, MALAT1, and Hotair—will be discussed in this section.

#### 4.2.1. H19

H19, which is highly expressed at the embryonic stage in mesodermal and endodermal tissues [155], and insulin-like growth factor II (IGF2) are reciprocally imprinted. After the early gestation period, H19 is solely expressed from the maternal-inherited allele whereas IGF2 is exclusively expressed from the paternal-inherited allele [156, 157]. Loss of IGF2 or H19 imprinting leads to IGF2 upregulation and subsequent H19 promoter hypermethylation, a phenomenon commonly found in cancers [158–160].

Studies have suggested that H19 possesses tumor-suppressor functions and have implicated chromatin insulator protein CCCTC-binding factor (CTCF) in methylating the promoter region of the* H19* gene [161, 162]. How H19 functions in cells has grown clearer with the introduction miRNAs [111, 163]. miR-675 is an intergenic miRNA embedded in the first exon of H19 and coexpressed with H19. H19/miR-675 negatively regulates insulin-like growth factor 1 receptor (IGF1R), nodal modulator 1 (NOMO1), and Twist proteins and suppresses TGF-*β*1/SMADs signaling [111–114, 164, 165]. Since TGF-*β*/SMAD signaling is a well-known EMT pathway, it is possible that H19/miR-675 plays a role in EMT regulation.

On the other hand, a cancer-promoting role of H19 has been suggested in colorectal and gastric cancers [115, 166]. In colorectal cancer, overexpressed H19 serves as a ceRNA that antagonizes miR-138 and miR-200a, leading to derepression of their endogenous targets, vimentin, ZEB1, and ZEB2 [115]. In addition, H19 was found to harbor several binding sites for Let-7 family miRNAs and act as a sponge that negatively regulates their activity [167]. Notably, expression of Let-7 miRNAs, which inhibit EMT by suppressing HMGA2, is frequently downregulated in cancers with a mesenchymal phenotype. Thus, H19 may exert an EMT-promoting function through its role as a miR-200 and Let-7 family sponge [116, 117].

These results suggest that the dual roles of H19 in cancer EMT regulation are deciphered by cellular context, in which different sets of miRNAs are involved in disease pathogenesis.

#### 4.2.2. MALAT1

MALAT1 (metastasis associated in lung adenocarcinoma transcript 1) has been reported to be a prognostic marker in several cancers, including lung, breast, pancreas, liver, colon, uterus, cervix, and prostate cancers [118]. In bladder cancer, TGF-*β*1 induces MALAT1 expression, whereas silencing of endogenous MALAT1 and its binding partner, SUZ12, suppresses TGF-*β*1-induced EMT [119]. In renal cancer, reciprocal crosstalk among MALAT1, miR-205, and EZH2 suppresses the expression of E-cadherin and enhances Wnt signaling activity, thereby promoting cancer metastasis [120]. EZH2 and SUZ12 are subunits of PRC2, which is responsible for the repressive histone 3 lysine 27 trimethylation (H3K27me3) chromatin modification. Previous studies have suggested that MALAT1 might be associated with the PRC2 complex and promote cancer EMT.

#### 4.2.3. Hotair

Hotair (Hox transcript antisense intergenic RNA) epigenetically regulates its target sequences by recruiting PRC2, which in turn results in gene silencing [168, 169]. Hotair upregulation was found to be a prognostic indicator of poor outcome in various types of cancers [121, 122]. In addition, Hotair was shown to be required for TGF-*β*-mediated EMT in colon cancer [170]. Hotair epigenetically silences miR-34 transcription, resulting in augmentation of C-Met and Snail expression [123]. It was also found that miR-141, an EMT suppressor, decreases the expression of Hotair through complementary binding and thereby inhibits its oncogenic functions [124]. miR-141 negatively regulates Hotair target genes, including* SNAIL*, the nonreceptor tyrosine kinase* ABL2*, and* PCDH10* (protocadherin 10). In addition, the expression levels of miR-141 and Hotair were found to be inversely correlated in renal cancer cells [124]. These results suggest that crosstalk between Hotair and miRNAs play important roles in cancer EMT regulation.

## 5. Conclusion

**Figure 3:**
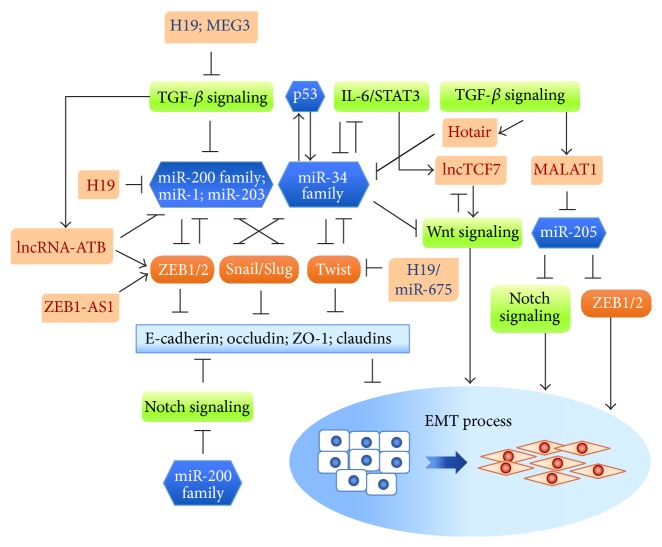
The molecular network composed of miRNAs/lncRNAs and EMT-TFs. miRNAs and lncRNAs form regulatory networks with EMT-TFs and EMT-associated signaling pathways that individually or cooperatively modulate EMT. EMT-suppressing miRNAs reciprocally suppress EMT-TFs and consequently downregulate the expression of epithelial markers. This negative feedback loop can be broken by TGF-*β* or IL-6/STAT3 signaling, p53, and lncRNAs (e.g., H19, lncRNA-ATB, and ZEB-AS1). Dysregulation of these miRNAs and lncRNAs may lead to tumor progression.

EMT is recognized as the first step of cancer metastasis—the main cause of cancer mortality. Cancer EMT is a tightly controlled pathological process. Multilayered regulatory elements, including proteins, miRNAs, and lncRNAs, are involved in the complex EMT regulatory networks through RNA-protein, RNA-miRNAs, and RNA-DNA interactions at pretranscriptional, posttranscriptional, and posttranslational levels. ncRNAs modulate epithelial plasticity by targeting different signaling pathways, EMT-TFs, and/or EMT-associated proteins. Several important reciprocal feedback loops, composed of ncRNAs and EMT-TFs, are involved in establishing flexible control over EMT and MET. Therefore, peeling back the mysteries surrounding ncRNAs in EMT regulation will be important in furthering advances in cancer therapy strategies (Figure 3).

## Figures and Tables

**Table 1:** miRNAs and other molecules involved in EMT.

miRNA	Expression levels in cancer	Upstream regulator	Known targets	References
miR-200 family	Breast cancer; prostate cancer	ZEB1/2, miR-22, Slug, GATA3, TGF-*β*, Foxf2	ZEB1/2, Slug, GATA3, Maml2/3, Foxf2	[69–72]

miR-1	Breast cancer; prostate cancer	ZEB1/2, miR-22, Slug, GATA3, TGF-*β*	ZEB1/2, Slug, GATA3, Maml2/3	[69–72]

miR-203	Breast cancer, pancreatic cancer	Slug, Snail, TGF-*β*	Bmi-1, Snail, ZEB1/2	[73, 74]

miR-34 family	Pancreatic stem cell, neuroblastoma	p53, epigenetic regulation	Snail, ZEB1	[75–77]

miR-9	Breast cancer	c-myc	E-cadherin, LIFR	[78, 79]

miR-135b	Colon cancer, NSCLC, HNSCC	Epigenetic regulation, NF-*κ*B, hypoxia	APC, LATS2, *β*-TrCP, NDR2, MOB1B	[80–82]

miR-210	Breast cancer	Hypoxia	E2F3, HOXA1, FGFLR1, EFNA3, PTP1B, VMP1	[83–85]

miR-103/107	CRC, breast cancer	Hypoxia	DAPK, KLF4, Dicer	[78, 86]

miR-10b	Breast cancer	Twist	HOXD-10	[87]

miR-21	NSCLC, CRC, breast cancer	TGF-*β*/BMP, HER2/neu, hypoxia	Pdcd4, TGFBR2, PTEN, TAp63	[88–92]

miR-205	Breast cancer	ΔNp63*α*	ZEB1/2, Jagged1	[69, 93, 94]

miR-23b	Colon cancer; bladder cancer	n/a	Src, ZEB1	[95–97]

miR-138	Ovarian cancer; HNSCC	n/a	SOX4, HIF-1*α*, vimentin	[98–100]

miR-7	Gastric cancer; breast cancer	WISP	IGF1R, Snail, SETDB1	[27, 101, 102]

HNSCC: head and neck squamous cell carcinoma; NSCLC: non-small-cell lung carcinoma; CRC: colorectal cancer; ZEB1/2: zinc-finger E-box binding homeobox 1/2; LIFR: leukemia inhibitory factor receptor alpha; APC: adenomatous polyposis coli; LATS2: large tumor-suppressor kinase 2; *β*-TrCP: beta-transducin repeat-containing protein; NDR2: nuclear-Dbf2-related 2; MOB1B: Mps one binder 1b; E2F3: E2F transcription factor 3; HOXA1: homeobox A1; FGFLR1: fibroblast growth factor receptor like-1; EFNA3: ephrin-A3; PTP1B: protein-tyrosine phosphatase 1B; VMP1: vacuole membrane protein 1; DAPK: death-associated protein kinase; KLF4: Krüppel-like factor 4; HOXD10: homeobox D10; Pdcd4: programmed cell death protein 4; TGFBR2: TGF beta receptor 2; PTEN: phosphatase and tensin homolog; WISP: WNT1-inducible signaling pathway protein 2; IGF1R: insulin-like growth factor 1 receptor; SETDB1: SET domain, bifurcated 1; n/a: not available.

**Table 2:** lncRNAs and EMT.

lncRNAs	Expression levels in cancer	Upstream regulator	Targets	References
ZEB1-AS1	HCC	n/a	ZEB1↑	[103]

lncRNA-ATB	HCC	TGF-*β*	ZEB1/2↑, IL-11↑, miR-200↓	[104]

lncRNA-HIT	Breast cancer	TGF-*β*	E-cadherin↓	[105]

MEG3	HCC		TGFBR1↑, TGFB2↑, SMAD2↑	[106]

lncRNA-Hh	Breast cancer	Twist	GAS1↑	[107]

lncTCF7	Liver cancer	IL-6	TCF↑ (Wnt signaling)	[108, 109]

treRNA	Breast cancer		E-cadherin↓	[110]

H19	n/a	CTCF	IGF1R↓, NOMO1↓, Twist↓, TGF- *β*1/SMAD↓, miR-138↓, miR-200↓, Let-7↓,	[111–117]

MALAT1	Lung cancer, breast cancer, liver cancer, prostate cancer, renal cell carcinoma	TGF-*β*1, EZH2↑	miR-205↓	[118–120]

Hotair	n/a	TGF-*β*1, miR-141	miR-34↓, miR-141↓, miR-7	[27, 121–124]

HCC: hepatocellular carcinoma; TGFBR1: transforming growth factor beta receptor 1; TGFB2: transforming growth factor beta 2; GAS1: growth arrest-specific 1; TCF: transcription factor; CTCF: CCCTC-binding factor; IGF1R: insulin-like growth factor 1 receptor; NOMO1: NODAL modulator 1; n/a: not available.
